# Microparticle image velocimetry approach to flow measurements in isolated contracting lymphatic vessels

**DOI:** 10.1117/1.JBO.21.2.025002

**Published:** 2016-02-01

**Authors:** Konstantinos N. Margaris, Zhanna Nepiyushchikh, David C. Zawieja, James Moore, Richard A. Black

**Affiliations:** a University of Strathclyde, Department of Biomedical Engineering, 106 Rottenrow, Glasgow G4 0NW, United Kingdom; b Georgia Institute of Technology, The George W. Woodruff School of Mechanical Engineering, Georgia Institute of Technology, Atlanta, Georgia 30332-0405, United States; c Texas A&M University, Department of Systems Biology and Translational Medicine, Health Science Center, Temple, Texas 77843-111, United States; d Imperial College London, Department of Bioengineering, Royal School of Mines, Exhibition Road, London SW7 2AZ, United Kingdom

**Keywords:** lymphatic system, lymphangion, microparticle image velocimetry, flow measurements, physiological flows, light-emitting diode

## Abstract

We describe the development of an optical flow visualization method for resolving the flow velocity vector field in lymphatic vessels *in vitro*. The aim is to develop an experimental protocol for accurately estimating flow parameters, such as flow rate and shear stresses, with high spatial and temporal resolution. Previous studies *in situ* have relied on lymphocytes as tracers, but their low density resulted in a reduced spatial resolution whereas the assumption that the flow was fully developed in order to determine the flow parameters of interest may not be valid, especially in the vicinity of the valves, where the flow is undoubtedly more complex. To overcome these issues, we have applied the time-resolved microparticle image velocimetry (μ-PIV) technique, a well-established method that can provide increased spatial and temporal resolution that this transient flow demands. To that end, we have developed a custom light source, utilizing high-power light-emitting diodes, and associated control and image processing software. This paper reports the performance of the system and the results of a series of preliminary experiments performed on vessels isolated from rat mesenteries, demonstrating, for the first time, the successful application of the μ-PIV technique in these vessels.

## 1 Introduction

The lymphatic system is a vital part of the circulatory and immune systems,^1^^,^^2^ and plays an important role in tissue fluid homeostasis and in combating infection. In contrast with the cardiovascular system, where the heart provides the necessary energy for blood flow, the lymphatic system relies on the active contraction of individual vessels and one-way valves to generate and sustain flow. It consists of lymphatic capillaries, collecting lymphatic vessels, and lymphoid organs. While the small lymphatic capillaries fill passively with interstitial fluid, the larger collecting lymphatic vessels actively contract to generate flow owing to local translumenal pressure and strain gradients within the extracellular matrix of the vessel wall. The ability of these vessels to contract originates from the smooth muscle cells (SMCs) that line the vessel wall, which are morphologically similar and exhibit similar molecular signaling and pace-making abilities as cardiac SMCs.^3^ Nonreturn, predominantly bicuspid, valves prevent the retrograde flow of lymphatic fluid. The portion of the vessel between two valves is referred to as a lymphangion. Individual lymphangions are arranged in networks to form the lymphatic vessels.

Reported attempts to measure the flow field inside these vessels are few in number. (The term flow field refers to the flow velocity vector field in the mid-plane of a lymphatic vessel. A vector field is a collection of vectors of given direction and magnitude. Each vector is assigned to a given point in space.^4^) In fact, excluding studies on lymph filtration, the only study to quantify temporal variations of flow velocity during contraction was that conducted by Dixon and coworkers,^5^^–^^7^ which was subsequently used by Kassis et al.^8^ These studies were performed *in situ*, in the exteriorized rat mesenteric area. Lymphocytes were used as tracers and images were acquired with a high-speed camera. An image correlation algorithm was used to measure the lymphocyte velocity.^9^ The authors inferred the flow velocity, volumetric flow rate, and wall shear stress (WSS) by assuming that lymphocytes follow the flow faithfully, and that the flow is laminar and fully developed. A major drawback of this approach is that the lymphocytes are relatively large in size (∼10  μm in diameter, whereas the vessel lumen diameter is, on average, 100  μm) and their density is usually low, reducing spatial resolution and, hence, the need to make assumptions with regard to the nature of the flow. Using lymphocytes as tracers limits the applicability to *in situ* measurements, and while such experiments may yield more physiologically relevant results with respect to an isolated *in vitro* preparation, the use of latter allows more control over factors that affect lymphatic function. Moreover, the latter study employed continuous wave (CW) illumination, which can significantly reduce the temporal resolution of an optical flow measurement system, rendering such a system less capable of resolving transient changes to flow velocity that may occur during the vessel contraction cycle.

Despite these drawbacks, these studies have been the most recent and only attempts in estimating fluid velocity and its temporal variations inside collecting lymphatic vessels. Several other attempts have been made to measure lymphatic flow rate in a variety of species; however, they are restricted to average flow rate measurements, tracking volumes of fluorescently tagged particles or fall under the flow cytometry methods; while very useful from a physiological or clinical perspective, these methods cannot give a detailed description of the local fluidic environment and especially WSSs, which is an important parameter that affects lymphatic function.

Onizuka et al. and Naito et al. implanted an ultrasound flow probe in sheep to measure the flow rate of the thoracic duct.^10^^,^^11^ The measured flow rates were three to six times greater than those measured in cannulated vessels, this fact being attributed to the invasive cannulation procedure. However, the authors did not clarify any effects the presence of the ultrasound probe had on the contraction of the thoracic duct. With this method, there is no *a priori* way to exclude the possibility of the vessel coming into contact with the probe as it contracts. The authors did not report measurements of lymph velocity or of WSSs.

McGeown et al. used a method that utilizes a transducer to measure lymph flow rate in conscious sheep by means of vessel cannulation.^12^ Lymph was allowed to accumulate on the transducer arm, and the weight of the fluid caused a tension reading on the transducer. The reading of tension was correlated with lymph volume leaving the cannulated vessel. This method, however, does not yield detailed flow field information.

Fedosov et al. developed an invasive flow cytometry method using a focused laser beam.^13^ The velocity and direction of lymphocytes was determined by cross-correlation of intensity fluctuations of the speckle field between two points. The method was applied *in vivo* in the rat mesenteric area; however, very limited results on measurements from different vessels were presented. Whether the method is able to measure a two-dimensional (2-D) flow field is unclear from the work published. According to Fedosov et al.^13^, the velocity measured is in relative units, and therefore, calibration was necessary with video microscopy. Using a focused laser beam also raises concern of potential damage of the lymphatic vessels.

Similar to the work by Fedosov et al., Kalchenko et al. developed a label-free *in vivo* laser speckle imaging for blood and lymph vessels.^14^ Although the method is able to demarcate lymphatic vessels, the long correlation/exposure times of their method (200 ms) render it incapable of measuring instantaneous flow velocity.

Galanzha et al. developed a photoacoustic flow cytometry method in order to count normal and abnormal immune cells in collecting lymphatic vessels in the rat mesentery and mouse ear, *in vivo*.^15^ Using such an approach, it is possible to measure the velocity of lymphocytes; however, it suffers the same drawbacks as in the work by Dixon et al., namely, lymphocytes are not ideal tracers because of their size, which makes it difficult to extract local flow velocity information.

The aforementioned studies target the flow in larger collecting lymphatic vessels. Swartz et al.^16^ and Berk et al.^17^ utilized fluorescent photobleaching to measure the flow in the lymphatic capillaries of the mouse tail. The method uses a fluorescent dye, instead of cells or particle tracers. Similarly, Fischer et al.^18^ used fluorescein isothiocyanate-dextran dye to measure flow velocity in lymphatic capillaries of the human skin. Although it is possible to measure velocity with fluorescent dyes, the lack of individual tracers reduces the spatial resolution and no spatially resolved flow fields were reported by the authors. Additionally, diffusion of the fluorescent dye induces experimental errors in the determination of fluid velocity.^19^

Noninvasive *in vivo* methods have also been utilized in lymph flow measurements in lymphatic collecting vessels.^20^^,^^21^ Vessels and nodes up to 3 cm below the surface were visualized with the use of near-infrared imaging with indocyanine green (ICG) tracer. However, longer wavelengths have an adverse impact on the intensity of light emitted, which necessitates the use of more sensitive sensors and reduces the spatial resolution. These studies in swine displayed the capability of the method to measure average velocity of tagged packets of ICG, but no spatially resolved information can be obtained, rendering the method unsuitable for accurately resolving the flow field in lymphatic vessels. Other noninvasive *in vivo* lymphangiography methods have been developed using an optical coherence tomography (OCT) approach or optical microangiography, a variant of OCT.^22^^,^^23^ OCT lymphangiography allows for label-free demarcation of lymphatic vessels and flow visualization. No quantitative information on flow in lymphatics was reported in these studies.

Motivated by the lack in available flow measurement methods, and in an attempt to overcome some of the limitations of previous work, we have employed the microparticle image velocimetry (μ-PIV) method.^24^ PIV involves seeding the flow with tracer particles and uses statistical methods to resolve the fluid velocity from consecutive images acquired at a given location. By increasing the tracer concentration, and reducing the particle diameter, the spatial resolution may be increased. Smaller particles, being less subject to the effects of gravity or inertia, exhibit excellent frequency response and settling times far in excess of the time scales of interest, with the result that they follow the flow more faithfully and provide a more accurate representation of the actual flow field. Moreover, by appropriate synchronization of light pulses and image acquisition, that is, the frame-straddling technique, the temporal resolution of such system can be significantly increased.^25^

With the above observations in mind, we have developed a low-cost light source, using high-power light-emitting diodes (LEDs) as well as associated synchronization software. The resulting images were processed with open-source code, which was extended to incorporate vessel wall tracking algorithms and image filters. The purpose of this paper is to describe the development of the experimental apparatus and to present preliminary analysis of data acquired *in vitro* from images of actual mesenteric lymphatic vessels and valves, thus demonstrating the applicability of μ-PIV in these vessels.

## 2 Material and Methods

### 2.1 Light-Emitting Diode Light Source Development

The LED light source utilizes high-power white and monochromatic LEDs (CBT-90 white/green, PT-120 green, and CBT-140 white, Luminus Devices).^26^ The driving electronics are from the same manufacturer (DK-136M development kit) and are capable of driving the LEDs with current pulses of up to 36 A. The pulse width time is adjustable from 2  μs to several milliseconds. The pulse separation time $\Delta t_{p}$ is also controllable and can be as low as 10  μs. Power is provided by a 650 W power supply (XP Power, Singapore). Control and synchronization of the source was implemented with LABView and a multifunction data acquisition device (National Instruments). A five-axes kinematic mount (EKSMA Optics, Lithuania) provided alignment of the LED with a quartz fiber-optic light guide, which delivered the light to an inverted microscope (Nikon, Zeiss). In order to increase the light collection efficiency of the LED/fiber-optic interface, several lens combinations were tested and the highest radiometric power output was obtained with an aspheric condenser lens with numerical aperture (NA) of 0.9.

The output of each LED was measured with a power meter (Thorlabs PM100A/S120VC), while a spectrometer (AVS-MC2000, Avantes BV, The Netherlands) was used to obtain the spectrum of the emitted light. The images were acquired with several high-speed cameras: a Photron SA-3 and MC-1 (Photron Inc.) were used during the development phase at Strathclyde University, whereas a Phantom V5.2 (Vision Research) was used at Texas A&M University, where the experiments in lymphatic vessels took place.

### 2.2 Microfluidic Experimental Setup

#### 2.2.1 Microparticle image velocimetry setup

**Fig. 1:**
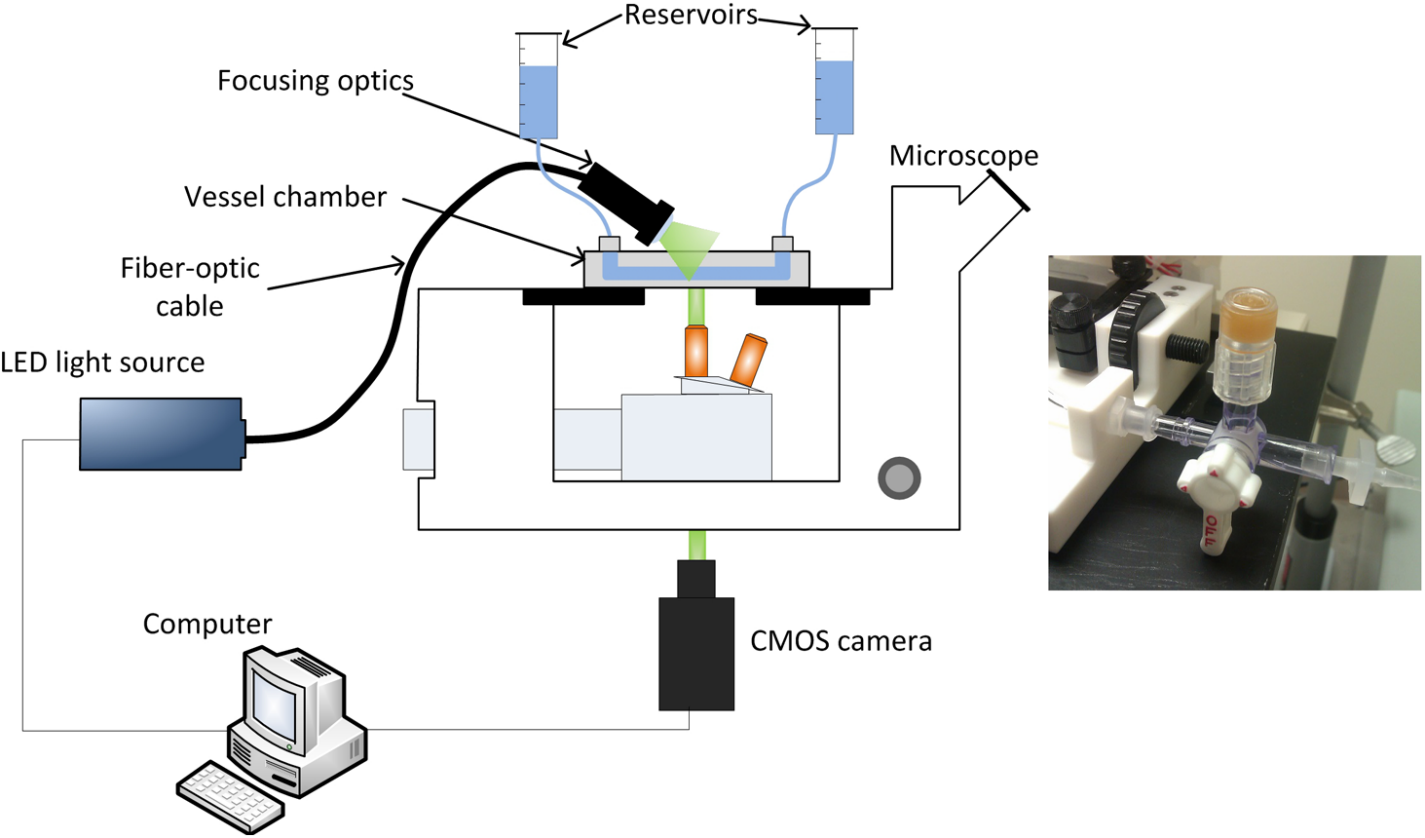
Experimental setup and camera light synchronization: (a) typical μ-PIV setup. A light source illuminates particles seeded in the flow, with high frequency, short-duration pulses, through a microscope objective. A camera captures images in synchrony with the light source, which are transferred to a computer for spatial cross-correlation analysis. Although fluorescent particles are commonly used in μ-PIV, in the current setup, nonfluorescent particles were used with the light being delivered from above and at an angle with respect to the specimen. Pressure was adjusted by changing the height of the inflow and outflow reservoirs. Image adapted from Ref. 28 with permission. (b) Injection port that was used to deliver particles. A three-way valve was used to isolate the vessel during particle injection for protection against pressure.

A μ-PIV setup was constructed around a Zeiss inverted microscope (20× magnification), following the guidelines available in the literature.^19^^,^^24^^,^^27^ The most significant difference with respect to a typical system [shown in Fig. 1(a)] is the means of illumination, which is explained in detail below. The flow was seeded with microparticles and the LED light source used provided suitable short burst pulses to capture the particle motion while minimizing streaking. Images were acquired by a CMOS camera (Vision Research, Phantom V5.2) and transferred to a computer for analysis. The field of view (FOV) of the CMOS sensor was 659×512  μm, which was sufficient to focus on a middle section or a valve, but not large enough to fit two adjacent lymphangions. Although the sensor FOV was smaller than the microscope objective FOV, the latter was not great enough to fit into view two adjacent lymphangions.

Pressure was adjusted by changing the height of the inflow and outflow reservoirs. Axial pressure gradient in this context is defined as $\Delta P_{\text{axial}}=P_{\text{out}}-P_{\text{in}}$. Therefore, when the inflow reservoir is raised higher than the outflow one, the pressure gradient is negative (favorable gradient) and drives the flow. In contrast, when the outflow reservoir is raised higher than the inflow one, $\Delta P_{\text{axial}}$ becomes positive (adverse gradient); forward flow, that is, flow along the direction of the vessel as allowed by the orientation of the one-way valves, cannot be maintained by the axial pressure gradient. The translumenal pressure $P_{\text{tr}}$ is the average value of the inflow and outflow pressures minus the external hydrostatic pressure exerted by the fluid column above the vessel (2 cm $H_{2}O$).

Nonfluorescent polystyrene 1  μm particles (density $\rho =1.05\;g/{\mathrm{cm}}^{3}$) were used. The particle response time is a function of the particle diameter $d_{p}$, particle density $\rho_{p}$, and the fluid viscosity μ, and is given in Eq. (1). This time, in water at 37 deg, is ∼0.08  μs, which is considered to be low enough for the flows under investigation in this study.^27^ Their settling time is also long: it would take ∼30  min for particles of 1  μm diameter to settle 50  μm, which is approximately the radius of rat mesenteric lymphatic vessels.^29^ This time is long enough compared to the time scales of the flow in question so that the effects of gravity may be safely ignored for the flows under investigation.

$$\tau_{p}=d_{p}^{2}\frac{\rho_{p}}{18\mu }.$$ (1)

The particle diameter on the camera sensor (particle image diameter) is important and influences the random error in determining the particle displacement between frames.^19^^,^^27^ The optimum particle image diameter that minimizes the random error corresponds to 2 to 4 pixels. With the current setup, the particle image diameter was 3.9 pixels, as estimated by the following equation:^30^

$$d_{\tau }=\sqrt{{\left(M\cdot d_{p}\right)}^{2}+{\left[2.44\cdot f_{\#}(M+1)\lambda \right]}^{2}},$$ (2)

where M is the microscope magnification, $f_{\#}=1/(2\mathrm{NA})$ is the f-number, NA=0.5 is the numerical aperture of the objective, λ is the wavelength of light, and $d_{p}$ is the physical particle diameter. This equation is in good agreement with the experimental results at 20× magnification.^31^

**Fig. 2:**
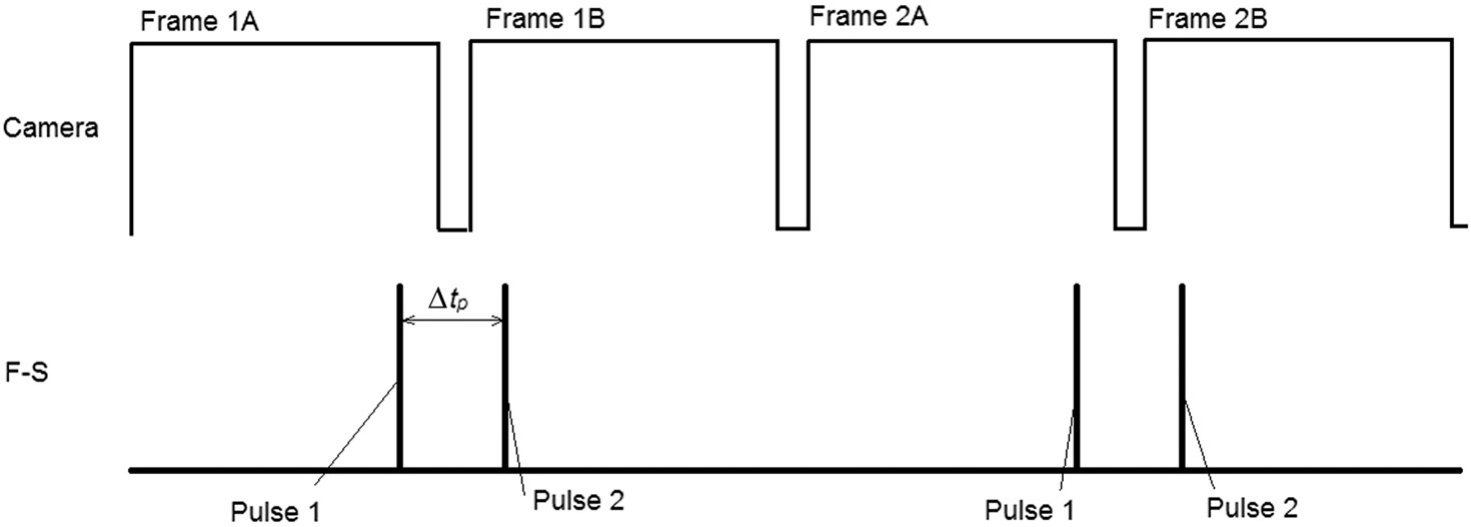
Example of frame straddling (F-S). The camera is set to record at a specific frame rate. In order to acquire one pair of images for PIV, the first light pulse is placed toward the end of the first frame (frame 1A) and the second light pulse at the beginning of the second frame (frame 1B) as to straddle the interframe time. With this approach, the time between frames is limited by the camera interframe time, or 2  μs in the current implementation. The next pair of frames (frames 2A/2B) must be acquired at half the camera fps in order to avoid double exposure.

Frame straddling is a method for increasing the temporal resolution of a PIV system and is illustrated in Fig. 2. The light pulses are placed in such a manner as to straddle the interframe time, which defines the limit of the temporal resolution. In our system, this time was 2  μs; hence, it was possible to have sufficient temporal resolution without increasing the frame rate of the camera; it also reduces the image memory storage requirements. In contrast, if frame straddling is not used, the light source can be synchronized to provide one pulse per camera exposure at the center of each frame. However, this constrains the temporal resolution to the camera frame rate. In other words, setting the camera at 250 fps, the temporal resolution becomes 1/250=4  ms, and to achieve a 2  μs resolution, the camera needs to be set at 500,000 fps. Using CW illumination has the same drawback too, and in addition, particle streaking may occur. Our research showed that even at adverse pressure gradients in lymphatics, the required temporal resolution can be as low as 1 ms (1000 fps); hence, the current implementation is capable of achieving this resolution without expending too much memory. The PIV pair of frames are acquired at half the camera frame rate, but modern cameras usually have high enough frame rate to compensate for the loss in acquisition rate. The camera frame rate was set to 10 to 500 fps and the pulse separation time adjusted so that the particle displacement was 5 to 10 pixels between frames. This is less than one quarter of a 64 pixels interrogation window and is considered to be a good choice in PIV experiments and often termed as the one-quarter rule (cf. Sec. 2.3). Since the expected flow velocity was not known, it was necessary to take successive measurement with varying pulse separation time. Pulse separation time ranged from 10  μs for flow at high negative pressure gradient up to 10 ms for flows at positive pressure gradient. The use of light pulses instead of CW illumination has additional advantages: it reduces the amount of light that the vessels are exposed to and reduces the cooling requirements of the LEDs.

**Fig. 3:**
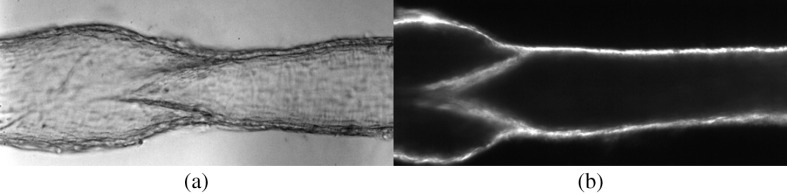
Image of a lymphatic vessel and valve at 20× magnification: (a) bright-field and (b) side-scattering illumination mode. Here, light was delivered from a fiber-optic cable positioned above and at an angle of ∼45  deg with respect to the microscope stage. The low numerical aperture of the objective used (M=20×, NA=0.5) ensured that stray light from the bottom wall was not significant.

A typical microscopic view of a single lymphatic vessel under two forms of illumination is presented in Fig. 3. When illuminated in bright-field mode via the epifluorescence module of the microscope [Fig. 3(a)], diffuse light scattered from the structures in and out of the focal plane is captured, reducing the effective contrast of the images. When illuminated from above (and at an angle) via the fiber-optic cable [Fig. 1(a)], however, the contrast of the images improves, with much of the light scattered from objects located within the focal plane of the microscope [Fig. 3(b)].

#### 2.2.2 Isolated lymphatic vessel preparation protocol

All experiments involving *ex vivo* lymphatic vessel preparations were carried out at the Texas A&M Health Science Center, Temple, Texas. The animal facilities used for these studies were accredited by the Association for the Assessment and Accreditation of Laboratory Animal Care, and adhered to the regulations, policies, and principles detailed in the Public Health Service Policy for the Humane Care and Use of Laboratory Animals (PHS Policy, 1996) and the U.S. Department of Agriculture’s Animal Welfare Regulations (Animal Welfare Act, AWA, 9CFR, 1985, 1992). All animal procedures performed for this study were reviewed and approved by the Texas A&M Institutional Animal Care and Use Committee.

Mesenteric lymphatic vessels and segments of the thoracic duct were isolated from anesthetized Sprague-Dawley rats and cannulated in a vessel chamber. The bath solution was an albumin-enriched physiological solution (APSS). The same liquid medium was used as solvent for the particle suspension. Details regarding the vessel isolation protocol can be found in the work of Gashev et al.^32^

Polystyrene microparticles of 1  μm diameter (Polysciences Europe GmbH, Germany) were either introduced into the upstream pipette with the vessel uncannulated to avoid damage, or via an injection port [Fig. 1(b)]. In the latter case, the vessel was cannulated and was isolated by a three-way valve to avoid damage during particle injection. Approximately 0.5 ml of particle solution was required; after initial trials, 0.5% weight-to-volume particle concentration was used in order to have 7 to 10 particles per interrogation window, which is an optimum value in PIV.^19^ Data on the refractive index of lymphatic tissue are difficult to find in the literature. Galanzha et al.^33^ report a value of 1.38 for rat mesenteric tissue, which is close to the refractive index of water (RI=1.33). Therefore, the major source of refractive index mismatch errors are likely to originate from the aperture on the vessel chamber bottom (RI=1.58) and the air objective used (RI=1). Temperature was regulated at physiological levels of 36 to 38°C.

### 2.3 Image Analysis

The image acquisition sequence generates a series of image pairs. Since the frame rate and pulse separation time are known, each pair of images may be spatially cross-correlated in order to calculate the velocity vectors of individual particles within the FOV. Briefly, a pair of frames is analyzed in each step. The images are divided into smaller interrogation windows (IW) (32×32 and 64×64  pixels in the present study). The IW from the two frames are cross-correlated and the resulting particle displacement divided by the pulse separation time to give the velocity vector associated with those particles within the IW. Other flow parameters may be derived from the flow velocity field. More information on PIV cross-correlation algorithms can be found in the literature.^19^^,^^27^

**Fig. 4:**
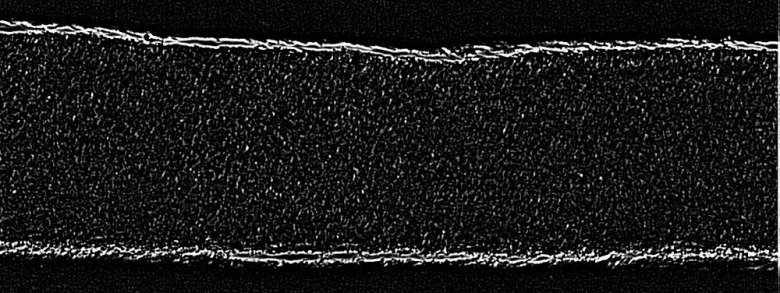
Image processed by a combination of unsharp and smoothing filters prior to PIV analysis.

Here the analysis was performed with the open-source MATLAB^®^ toolbox PIVlab (version 1.32).^34^ Established image-processing macros for background subtraction and image enhancements were implemented,^35^^–^^37^ and image overlapping algorithms were employed to compensate for low seeding density in the case of steady flows.^38^ The filter that yielded the optimum results, in terms of noise reduction, was that developed by Gui et al.^35^ Figure 4 shows an image of a lymphatic vessel containing particles that have been processed with this filter.

In PIV, postprocessing of the resulting vector field is necessary, in order to remove outliers from the results. PIVlab implements a number of postprocessing options that are common in PIV analysis. Here, a novel automatic and robust method developed by Garcia^39^ was used. This method is a penalized least square approach that allows for automatic smoothing of data in one or higher dimensions. Smoothing is carried out by direct cosine transformation. The degree of smoothing is determined by a minimization algorithm. The algorithm also deals efficiently with outliers or missing data. Further details can be found in the original publications.^39^^,^^40^

**Fig. 5:**
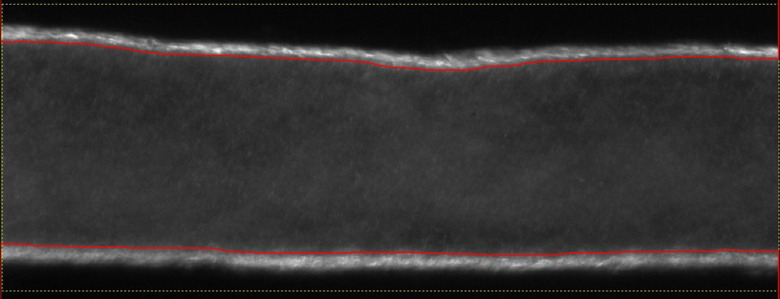
Example of vessel wall detection. The red line denotes the detected vessel wall edge using Canny edge detection.

Vessel contraction adds an additional complexity to the problem: that of detecting the vessel wall in order to create a mask and removing the wall during cross-correlation. (In some cases, the vessel may also rotate or move perpendicular to the image plane during contraction. However, this can be avoided with careful vessel cannulation and ligation.) While manual masking is possible, it hinders the analysis of large data sets. A vessel wall detection algorithm was developed (Fig. 5 shows a representative result), based on Canny edge detection.^41^ Prior to applying the Canny edge detection, the images were preprocessed, either by means of thresholding or by the application of mask filters.^42^

### 2.4 Wall Shear Stress Estimation

The WSS was estimated by a curve-fitting procedure whereby a third-order polynomial was fitted to the four velocity vectors derived from IW adjacent to the vessel wall. The WSSs at a given axial location was taken to be the average of the values determined at opposite walls, in order to account for asymmetry in the velocity profile. (In this implementation, the velocity from the PIV analysis at the wall was used; this is generally nonzero, and although this deviates from the no-slip velocity condition at the wall, it has been shown to yield more accurate results than replacing the velocity value at the wall with zero.^43^)

Since the experiments were carried out at physiological temperatures of 36 to 38°C and APSS is an aqueous solution, it was considered reasonable to assume that APSS has the same properties as pure water at the same temperatures (density $\rho =993\;\mathrm{kg}/m^{3}$, dynamic viscosity $\mu =6.78\times 10^{-4}\;\mathrm{Pa}\cdot s$).

## 3 Results

### 3.1 Light-Emitting Diode Light Source

**Fig. 6:**
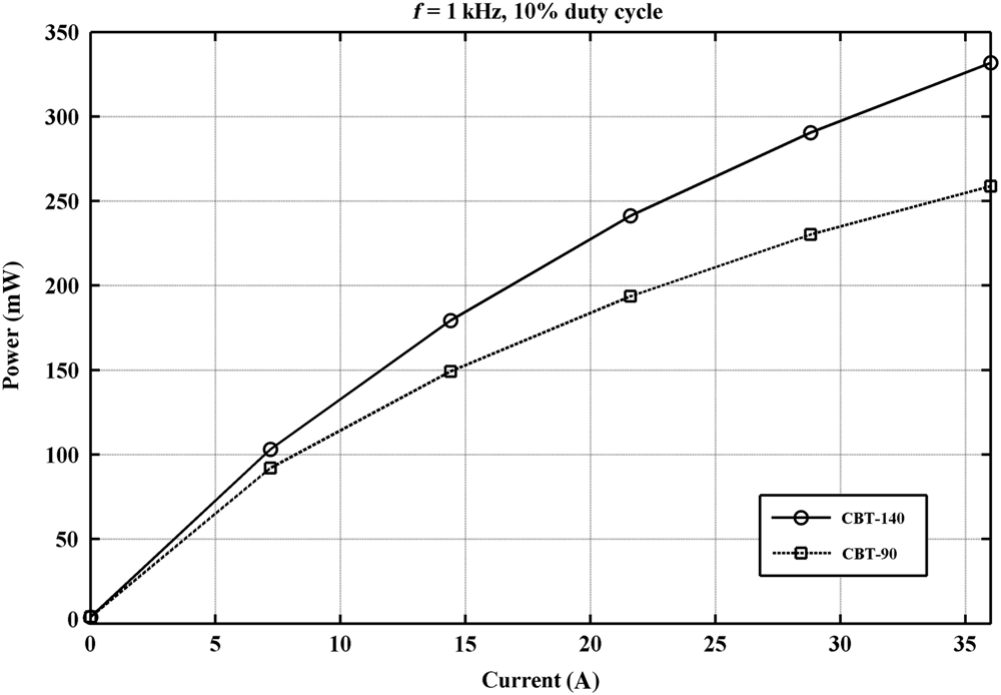
Power output of two white LEDs by Luminus Devices, CBT-90 and CBT-140. The measurement was performed at the exit of a 6 ft fiber-optic light guide. Despite the considerable losses at the LED/fiber-optic bundle interface, the power output is sufficient for optical flow diagnostic methods, such as the one presented here.

The LED light source was characterized in terms of LED power output pulsed mode (PM) illumination at different levels of input current, pulse frequency, and duty cycles. Figure 6 compares the energy per pulse of different LEDs from the same manufacturer, measured at a frequency of 1 kHz with 100  μs pulse duration. (Generally, the optical power output required for such experiments is not reported in the literature; hence, we show the indicative measurement in Fig. 6, which may be of use to researchers planning similar experiments.) During experiments in lymphatic vessels, our system could yield good contrast with 50  μs pulse duration; hence, the power per pulse was half the one reported in Fig. 6.

The driver circuit can pulse the LED at frequencies of up to 3 kHz, with up to 36 A current pulses. The time interval between two successive pulses can be as short as 5  μs, which, taking into account the one-quarter rule in PIV,^44^ corresponds to velocities in excess of 100  mm/s at 20× magnification.^45^ While the mean flow velocity throughout the lymphatic system is generally lower than this value (under positive or zero pressure gradient, the maximum velocity we observed was ∼10  mm/s), under negative pressure gradient of $-5\;\mathrm{cm}\;H_{2}O$ it can reach 50  mm/s.^46^

### 3.2 Microparticle Image Velocimetry System Performance

In terms of spatial resolution, using 64 pixels IW with 75% overlap yields ∼20 velocity vectors along the diameter of a fully distended vessel. This number is reduced to ∼10 vectors at the end systolic diameter.

A detailed *a priori* analysis of the measurement uncertainty in PIV is generally not possible. Therefore, an *a posteriori* analysis has to be performed instead. The analysis, which included the effects of the optical setup, random errors due to the PIV algorithm and Brownian motion, showed that the cumulative, measurement uncertainty, estimated at the maximum velocity at the center of the vessels, remained below 6% over the entire range of experiments performed.^46^ The minimum resolvable velocity is given by the root mean square error in estimating the particle displacement between frames and may be estimated by the following equation:^47^

$$\sigma_{u}=\frac{c_{\tau }d_{\tau }}{M\Delta t}.$$ (3)

For the current system, the particle image diameter, $d_{\tau }$, was equivalent to 3.9 pixels at M=20× magnification. The constant $c_{\tau }$ is equal to 1 to 10%. Assuming that $c_{\tau }=5\%$ and Δt ranges from 10 ms to 50  μs, the resulting minimum resolvable velocity is 0.56 to 111.74  μm/s (the calibration factor for the system was 0.573  μm per pixel).

**Fig. 7:**
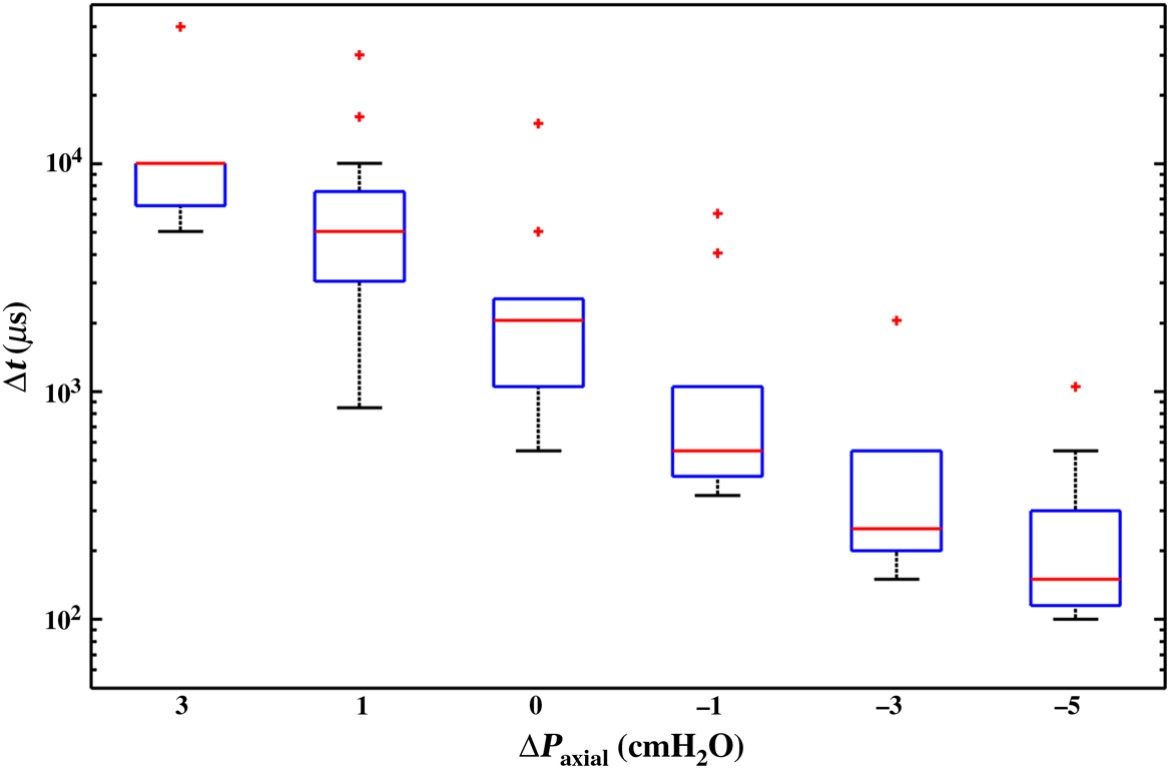
Temporal resolution with respect to axial pressure gradient. A positive (adverse) axial pressure gradient indicates that the outflow reservoir is raised higher than the inflow one and flow is driven by the active vessel contraction. At a negative (favorable) axial pressure gradient, flow occurs without the need of vessel contraction.

As one would expect, the greater the favorable pressure gradient, the greater is the corresponding flow velocity. It follows that the temporal resolution need not be so great to resolve the flow velocity, as is apparent in Fig. 7. The relatively large deviation of data from the mean at each value of axial pressure gradient is due to the natural variation of vessel diameter from lymphangion to lymphangion of the same isolated vessel, and within the same lymphangion. It could also be attributed to the uncertainty in inflow and outflow pressure adjustment. Another potential source of this variation is the cannulation micropipette resistance. For this reason, the experiments on lymphatic vessels were carried out with the same set of resistance-matched micropipettes.

Figure 7 again shows the benefit of employing frame straddling to measure the instantaneous velocity inside lymphangions. The median Δt is 10 ms for $\Delta P_{\text{axial}}=3\;\mathrm{cm}\;H_{2}O$. Without frame straddling, a camera working at 100 fps would have been necessary. At this $\Delta P_{\text{axial}}$, this is not very restrictive, but with frame straddling, the velocity can be measured with much lower fps, resulting in less data to process for a certain measurement duration, or allowing PIV measurements for longer periods of time. At the other extreme, at high negative axial pressure gradients, 10k fps would be necessary without frame straddling, resulting in a large number of pairs of frames and reducing the measurement duration due to camera memory limitations. Thus, the method employed by Dixon and coworkers^9^ would be challenged at high negative axial pressure gradients since even at zero gradient, a camera with 1000 to 2000 fps capability is generally required. On the other hand, this is not a concern with the current method, as the temporal resolution is mainly limited by camera interframe time, which, for modern PIV cameras, is of the order of nanoseconds.

### 3.3 Lymphatic Valves

**Fig. 8:**
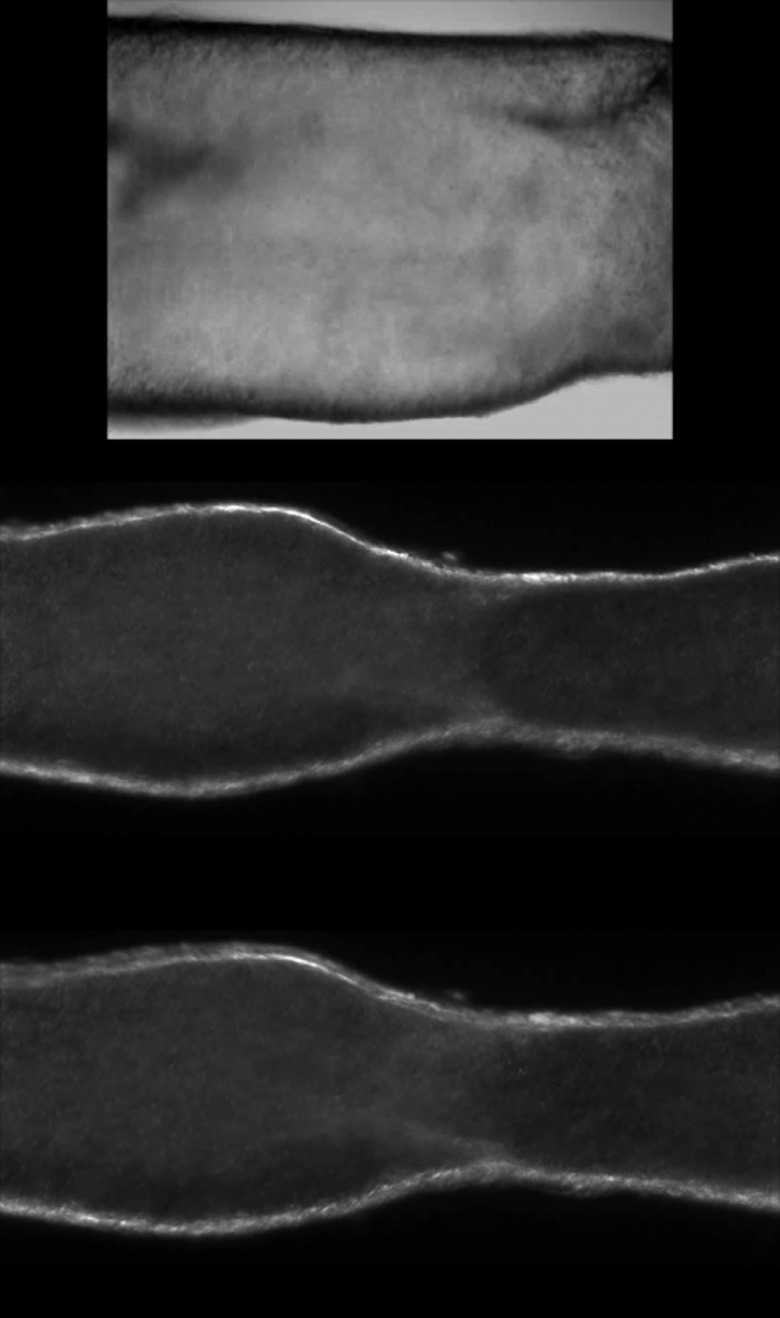
Videos around valve leaflets: (a) thoracic duct valve at negative pressure gradient (Video 1, MP4, 16.7 MB) [URL: https://doi.org/10.1117/1.JBO.21.02.025002.1], (b) mesenteric valve at negative pressure gradient (Video 2, MP4, 6.2 MB) [URL: https://doi.org/10.1117/1.JBO.21.02.025002.2], and (c) mesenteric valve at positive pressure gradient (Video 3, MP4, 5.7 MB) [URL: https://doi.org/10.1117/1.JBO.21.02.025002.3].

Eddies around the leaflets of a thoracic duct valve were recorded with the μ-PIV system. Video 1 [Fig. 8(a)] of the accompanying multimedia material shows such an eddy, recorded with CW illumination at 1000 fps. The translumenal pressure was set at 2 cm $H_{2}O$, and the flow was driven by an axial pressure gradient of $-1\;\mathrm{cm}\;H_{2}O$. These eddies were first reported by Florey in 1927 in guinea pig lymphatic vessels.^48^ Eddies around the valve leaflets were also observed in mesenteric lymphatic vessels. Videos 2 [Fig. 8(b)] and 3 [Fig. 8(c)] show recirculation at negative ($-0.5\;\mathrm{cm}\;H_{2}O$) and positive ($+0.5\;\mathrm{cm}\;H_{2}O$) pressure gradient, respectively, at 2 cm $H_{2}O$ translumenal pressure, recorded at 500 fps in PM illumination.

**Fig. 9:**
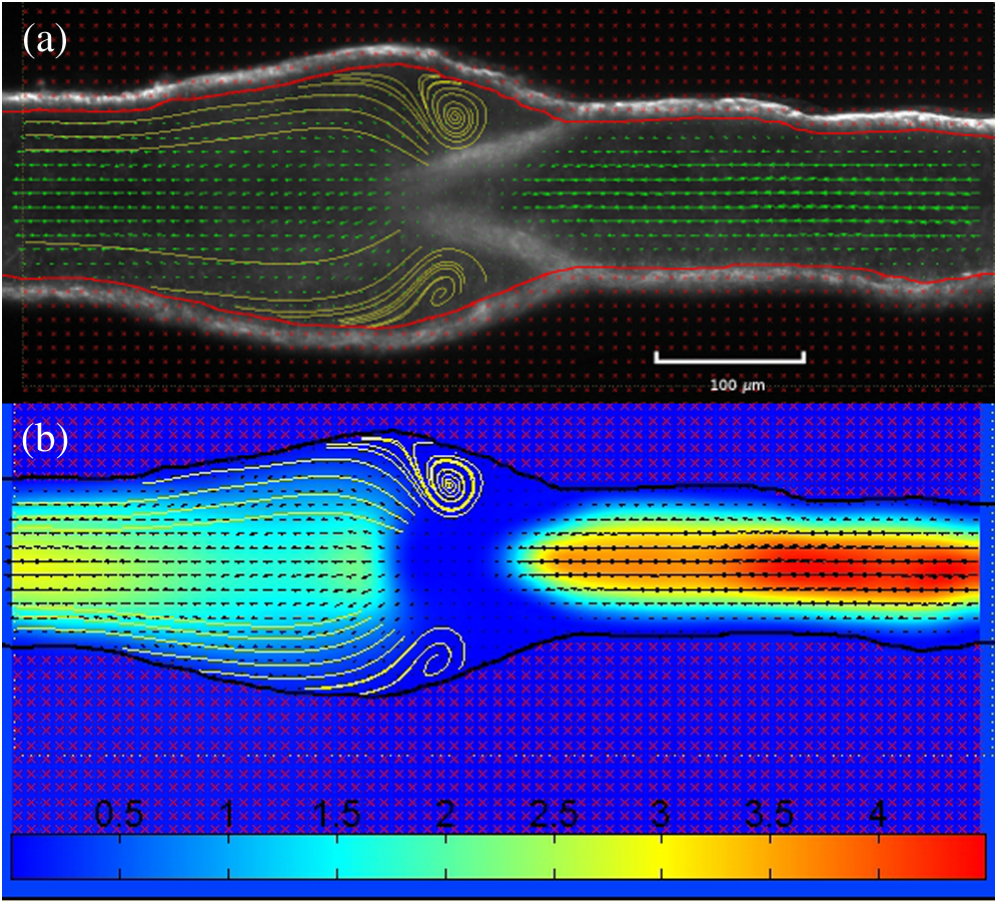
Eddies forming around valve leaflets at an axial pressure gradient of $-1\;\mathrm{cm}\;H_{2}O$ at a translumenal pressure of 1 cm $H_{2}O$. This measurement was performed in a noncontracting vessel, and therefore, the flow was at steady state. Approximately 100 pairs of frames were acquired and the results averaged. Only one third of the velocity vectors are plotted for clarity. (a) Streamlines identifying recirculation around the lymphatic valve and (b) colormap of the velocity magnitude. The scale is in mm/s. The Reynolds number calculated upstream from the valve is ∼0.45.

The PIV system was also able to quantitatively resolve secondary flows in the regions behind valve leaflets (Fig. 9). The spatial resolution of PIV provides means of visualizing and quantifying these flow structures with much greater detail than other flow diagnostic techniques. On closer inspection of Fig. 9(b), however, the velocity inside the valve appears to be zero; clearly, this cannot be the case. Arguably, this result may be due to geometrical asymmetries in the valve region and out-of-plane particle motion. The microscope optics were focused so that the image plane coincided with the mid-plane of the straight vessel segment close to the valve; the plane of maximum velocity between the valve leaflets may not necessarily coincide with the latter. Out-of-plane particle motion and noise from the valve leaflets may also be additional factors affecting the measurement in this region.

### 3.4 Lymphatic Vessels

The system developed was also able to measure the transient flow rate throughout the contraction cycle over a wide range of hydrodynamic conditions. The image processing time was ∼1  s for the vessel wall detection and 2 s for the cross-correlation per pair of frame on a laptop with an Intel Core 2 Duo T9500 CPU.

**Fig. 10:**
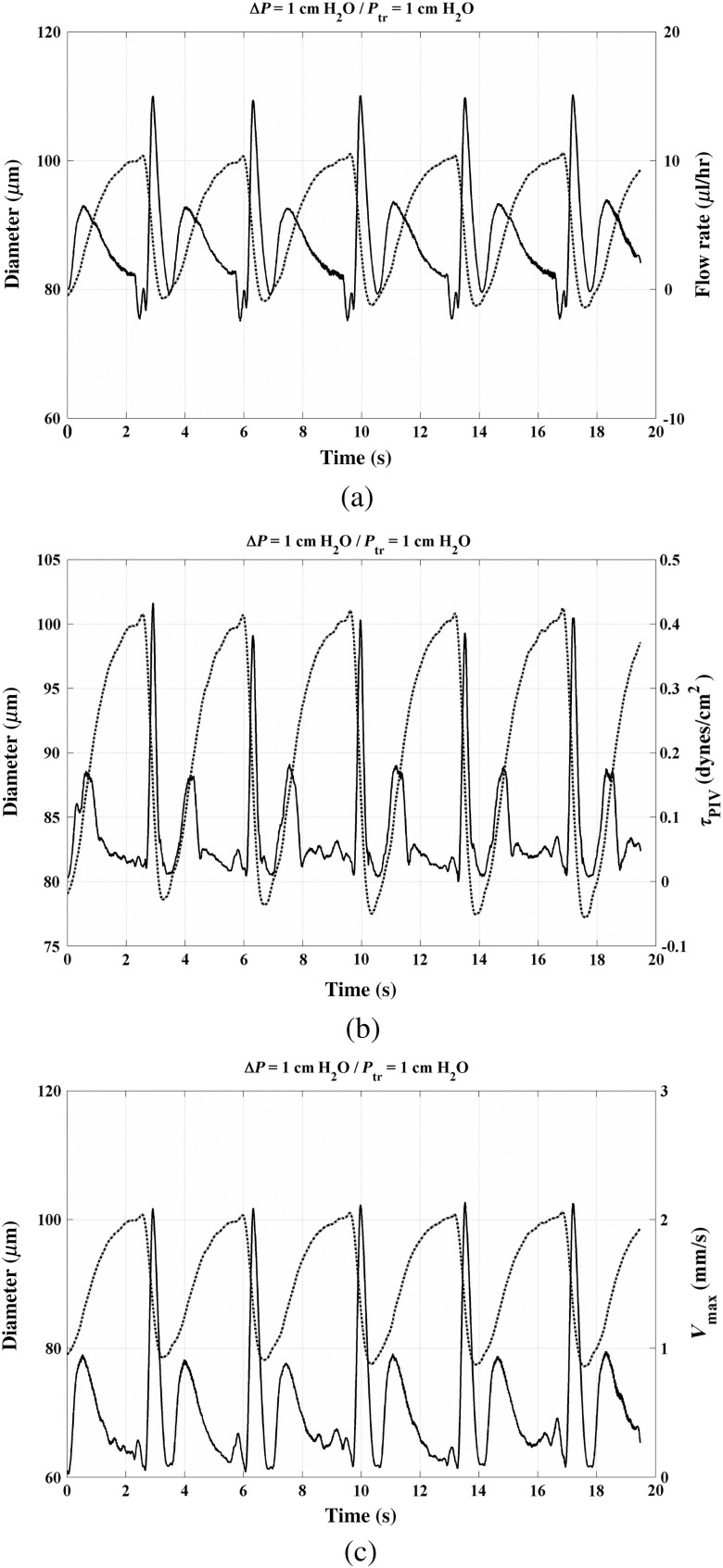
Temporal variation of flow parameters and vessel diameter at adverse axial pressure gradient: (a) flow rate (solid line) and vessel diameter (dotted line), (b) maximum velocity (solid line) and diameter (dotted line), and (c) PIV derived WSS (solid line) and diameter (dotted line).

The results of a flow measurement in a lymphatic vessel containing three valves and pumping against an adverse pressure gradient (the output reservoir is higher than the input one) are shown in Fig. 10 in solid lines; the dash-dot line depicts the vessel diameter. In this case, five contraction cycles occur in 20 s; thus, the contraction frequency is ∼15 beats per minute. Flow rate, calculated by integrating the velocity profile at a cross-section using the trapezoidal rule and assuming that the vessel has a circular cross-section, is shown in Fig. 10(a). Most of the positive flow (area under the solid curve) appear to take place during vessel distension rather than contraction. This observation demonstrates that flow in any lymphangion depends on the contractile status of the upstream and downstream lymphangions. Our preliminary results suggest that this is not a general case, but occurs frequently. The maximum velocity and WSS are shown in Figs. 10(b) and 10(c), respectively.

When a favorable axial pressure gradient is imposed, positive flow occurs when the vessel is distended, whereas lymphatic contraction reduces the flow rate, owing to the increase in vessel resistance as a result of vessel diameter reduction. Video 2 is of a lymphatic valve at favorable pressure gradient of $-0.5\;\mathrm{cm}\;H_{2}O$. As the vessel contracts, flow stops. This video also demonstrates that, with appropriate cannulation of the vessel, it is possible to keep the particles at the mid-plane of the lymphangion in focus; occasionally, a vessel may move so that the particles go out of focus and it is necessary to attempt a recannulation.

**Fig. 11:**
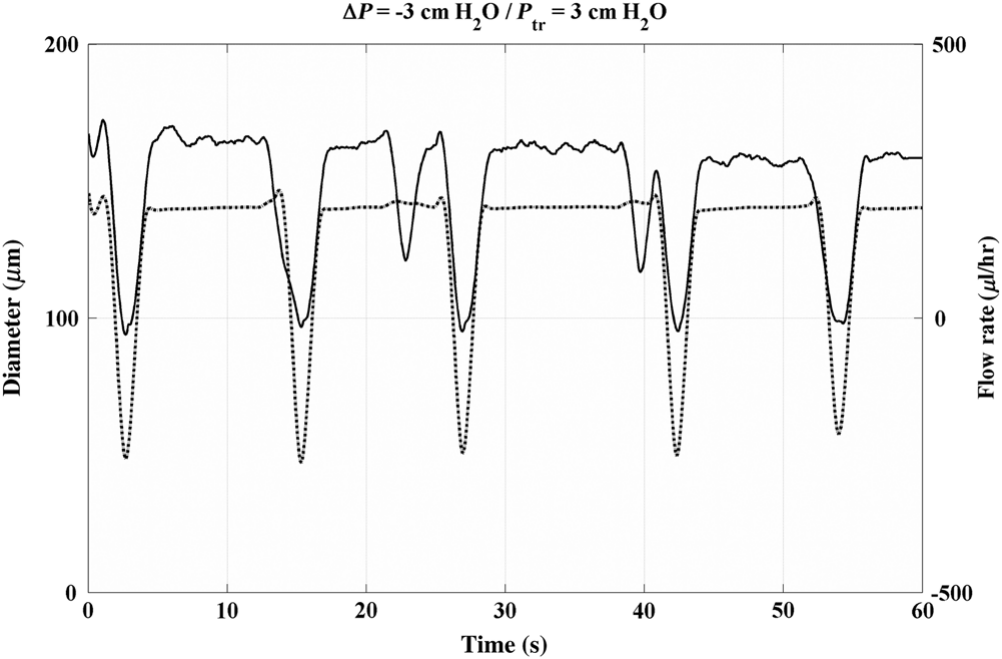
Temporal variation of flow rate (solid line) and vessel diameter (dotted line) at favorable axial pressure gradient.

Another example is shown in Fig. 11, which shows the flow rate and diameter tracings at the middle of a straight segment of a lymphatic vessel with three valves, during imposed favorable pressure gradient of $-1.0\;\mathrm{cm}\;H_{2}O$. In addition, it is interesting that a sudden drop in flow rate is observed, but the vessel remains at its end diastolic diameter at the flow rate drop time. This indicates an increase in flow resistance somewhere along the vessel that may be attributed to an out-of-phase contraction of an upstream or downstream lymphangion that is outside the FOV of the experiment. Again, as is evident in Fig. 11, the sudden drop in flow rate demonstrates that the flow in a lymphangion may be affected by the contraction state of upstream and downstream lymphangions.

## 4 Discussion

LEDs offer a versatile, low-cost, safer alternative source of illumination to lasers for microfluidic flow measurements. The use of LED μ-PIV systems in physiological flow studies is relatively novel, and their potential is yet to be fully realized. The previous study by Willert et al.^49^ showed that the driving current can be increased dramatically, without damaging the LEDs. Although the driving electronics limited the maximum current to 36 A, the development of custom electronics that can pulse the LEDs with currents in the range of 200 A will enable the use of even shorter-duration pulses and allow the measurements of even faster flows.

The apparatus and results of the present study are promising and demonstrate, for the first time, the feasibility of the μ-PIV technique to quantify the 2-D flow field within contracting lymphatic vessels. The system described in this report represents a significant improvement in terms of spatial and temporal resolution compared to previous work. At 20× magnification, the system is capable of measuring velocities in excess of 100  mm/s (lower at higher magnification).^45^ It may seem counterintuitive that lymphatic flow velocity can reach such values; however, we have observed velocities in the range of 50  mm/s at high negative axial pressure gradient (results not shown). Even for slower velocities, the present implementation with frame straddling requires less memory for image storage and allows longer image acquisition times with the same available camera memory.

The likelihood of thermal damage is also reduced with respect to a laser-based system. In fact, our results indicate that the likelihood of significant damage to the vessel caused by the LED is minimal. In our preparations, the vessels attained a physiologic rate of contraction of 7.4 contractions per minute (mean value $7.4\;\min^{-1}$, standard deviation $2.9\;\min^{-1}$, n=11 vessels). The measurements were performed for no more than 60 s at a time in any case, owing to camera memory limitations, with short-duration light pulses; a considerable amount of light is also lost in the optical system or absorbed from the water bath. We were able to keep the vessels functioning for up to 4 h, which corresponds to other experiments with CW illumination.

As an *in vitro* method, it allows measurements under a controlled environment; flow can be measured under varied hydrodynamic conditions as well as under constant ones with the additional chemical or electrical stimuli. This is expected to create new insight into lymphatic flow.

It may also be feasible to extend the method to *in vivo* flow measurements, provided there is optical access to the vessels. More work will be necessary to that end however, as an appropriate route of particle administration needs to be established. In addition, LEDs are also a cold light source, which may prevent dehydration or thermally induced damage to the vessels. The lower phototoxicity of pulsed LED illumination compared to conventional light sources (e.g., mercury, halogen) is why the latter sources are being superseded.^50^ Thus, μ-PIV has the potential to become a valuable tool for studying the lymphatic system and may also provide experimental validation of computational models that have recently appeared in the literature.^51^^,^^52^

The system proved capable of revealing flow patterns around valves, which was previously only feasible with computational fluid dynamics tools.^51^ However, this is a more challenging measurement than flow in straight segments. Flow around and inside valves is of interest, as it has been shown that the endothelial cells produce nitric oxide (NO), a substance that affects SMC contractility. It is likely that there is a shear dependent mechanism that influences NO production, which varies locally in lymphatic vessels.^51^^,^^53^ In order to quantify the complex flow patterns present around valves, the apparatus may be extended to allow three-dimensional (3-D) characterization of the flow field. Several different approaches to 3-D μ-PIV exist, such as stereoscopic, holographic, and aberration based imaging. For a review of 3-D implementations, the reader is referred to the review by Cierpka and Kaehler.^45^

One drawback of the current setup, in fact of all μ-PIV equipment, is the restricted FOV of the available microscope objectives. The magnification of the objective employed in this study represents the optimal magnification for flow measurement based on the vessel average diameter of ∼100  μm. Unfortunately, the resulting FOV at 20× magnification does now allow for simultaneous imaging of adjacent upstream/downstream lymphangions, which may be contracting out of phase with respect to the lymphangion under observation. Reducing the magnification will of course increase the FOV but at the expense of spatial resolution. Larger-diameter particles may be needed in such circumstances, which may give rise to issues depending on their settling velocity. Although the phase relationships of contraction between adjacent lymphangions and the effect on the flow cannot be studied with the current implementation, the use of microelectromechanical deformable mirrors may offer a solution to this problem.^54^ Customized confocal microscope designs with wider FOV have recently appeared in the literature, which may offer a potential solution to this problem.^55^

The present implementation of WSS estimation assumes that the normal to the vessel wall is orthogonal to the main flow direction. This is not always the case with lymphatic vessels during contraction, and the fact that the vessel wall may not be straight. An improved approach would need to determine the normal vector at the wall location where WSS are to be computed and derive the velocity gradient with respect to this normal direction. This approach was used by Poelma and coworkers,^56^ but was not implemented here and is left as future work.

## 5 Conclusions

The preliminary data presented in this paper demonstrate the practical application of μ-PIV in measuring the flow field inside contracting lymphatic vessels with greater spatial and temporal resolution than has been achieved to date, and at far lower cost than equivalent laser-based systems. Nevertheless, the current system does have some limitations in terms of its hardware and software implementation. The hardware implementation limits the FOV, and the 2-D nature of the measurement limits the applicability of the measurement in the valve region; however, we are confident that these limitations may be overcome with appropriate improvements to hardware and software.

The present study has extended the applicability of μ-PIV to lymphatic flow measurements *in vitro*. An *in vitro* method has an advantage over *in situ* or *in vivo* methods in the fact that external stimuli, such as pressure or chemical environment, may be accurately controlled and their effect of flow may be derived. In contrast, the obvious disadvantage lies in the fact that it is not possible to measure flow in *in vivo* conditions at present. Further work is needed in finding a route of particle administration at sufficient concentration, potentially in a lymph node. If successful, the method has the potential to permit *in situ* measurement. Accurate measurement of the flow field in these vessels will provide insight into their function by providing a means of quantifying the fluidic environment and correlating its effect with changes in contractile behavior.
